# Recurrent Retroperitoneal Lymphatic Malformation in a Pediatric Patient: A Case Report

**DOI:** 10.7759/cureus.30846

**Published:** 2022-10-29

**Authors:** John Ciubuc, Tyler Morgan, Dylan Murray, Richard Murray

**Affiliations:** 1 Department of Surgery, Texas Tech University Health Sciences Center, Amarillo, USA; 2 Department of Pathology, University College Dublin, Dublin, IRL; 3 Department of Radiology, Northwest Texas Healthcare System, Amarillo, USA

**Keywords:** lymphatic malformation, retroperitoneal lymphatic malformation, pediatric, body ct, body mri, nonspecific abdominal pain, conservative and surgical treatment, retroperitoneal, lymphangioma, recurrent

## Abstract

Retroperitoneal lymphatic malformations are rare, benign, cystic tumors of the lymphatic system, accounting for 1% of all lymphatic malformation manifestations. Lymphatic malformations are often asymptomatic, but may clinically present with abdominal pain and a palpable mass. Initial diagnostic workup is challenging due to the difficulty of differentiating from masses that may arise from the pancreas, liver, and kidney. This report describes a recurrent retroperitoneal lymphatic malformation in a 15-year-old male. The patient presented with abdominal pain and radiological imaging demonstrated abdominal fluid collection. Following conservative management using aspiration, the patient presented three months later with recurrent abdominal pain. Radiological imaging identified a large thin-walled cystic lesion in the right hemiabdomen containing minimal thin internal septations. Histological analysis confirmed the lymphatic malformation following computed tomography-guided biopsy. The patient underwent aspiration and was referred for outpatient sclerotherapy to prevent future abdominal fluid collection. This case highlights conservative management of recurrent retroperitoneal lymphatic malformation, both on the initial and subsequent presentation.

## Introduction

Lymphatic malformations are rare, benign malformations of lymphatic tissues that predominately arise in the pediatric population [1-3]. The cervical (75%) and axillary (20%) regions are frequently involved, with uncommon involvement observed with the rest of the body (5%) [1-4]. Retroperitoneal involvement is a remarkably rare manifestation (<1.0%) of lymphatic malformations and is normally an incidental finding identified on abdominal imaging or surgical exploration [1,2,4]. The recurrence frequency for lymphatic malformations is low following complete resection (7%), but it is significantly higher following partial resection or conservative management (50%) [3,4].

Lymphatic malformation presentation can be markedly varied with cystic, cavernous, or capillary morphology with either uni- or multiseptate appearances [4,5]. Despite having varied anatomical manifestations, most retroperitoneal lymphatic malformations are asymptomatic. Early clinical symptoms are nonspecific, including abdominal or back pain, hematuria, fatigue, fever, and weight loss [4,5]. These symptoms may occur secondary to neighboring structure encroachment if the tumor mass is enlarged, as seen in the patient described here.

## Case presentation

The patient was a 15-year-old male with no relevant past medical history, who presented to the emergency department with right-sided abdominal pain of one-day duration. He characterized the pain as being gradual and sharp, of moderate intensity, with exacerbating factors including positional changes and coughing. The patient denied previous trauma with no history of nausea, vomiting, diarrhea, constipation, weight changes, or fever. Physical examination revealed a temperature of 37.1°C, a heart rate of 62 beats/minute, a respiration rate of 18 times/minute, a systolic and diastolic blood pressure of 122/71 mmHg, and a mild right lower quadrant abdominal tenderness with no masses palpated. Laboratory results were mostly unrevealing, with notable exceptions including a slightly elevated white blood cell count of 12,100/mm3 (normal range: 4,500-11,000/mm3) with a left shift of 79.6%, and slightly elevated total bilirubin of 1.1 mg/dL (normal range 0.1-1.0 mg/dL) (Table 1).

**Table 1:** First admission vital signs and laboratory values.

Vital signs and laboratory values on the first admission		
Vital signs	Patient measurements	Normal range
Temperature	37.1°C	36.1°C-37.2°C
Heart rate	62 beats/minute	60-100 beats/minute
Respiratory rate	18 times/minute	12-20 times/minute
Systolic blood pressure	122 mmHg	90-120 mmHg
Diastolic blood pressure	71 mmHg	50-80 mmHg
Notable laboratory values		
White blood cell count	12,100/mm3	4,500-11,000/mm3
Neutrophil percentage	79.60%	32-58%
Absolute neutrophil count	9,600/mm3	2,500-6,000/mm3
Total bilirubin	1.1 mg/dL	0.1-1.0 mg/dL

**Figure 1:**
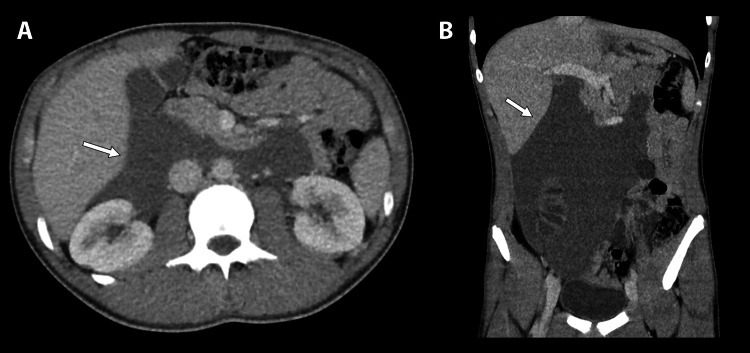
(A) Axial contrast-enhanced CT image demonstrates intermediate density free-flowing fluid in the right abdomen. The fluid displaces the pancreatic head anteriorly. (B) Coronal contrast-enhanced CT image demonstrates the extent of the free-flowing fluid throughout the abdominal cavity. Involvement includes the right paracolic gutter extending to the dome of the bladder.

A computed tomography (CT) scan of the abdomen and pelvis with contrast revealed a complex intermediate-density fluid collection within the right abdomen (Figure 1). The fluid collection extended from the right paracolic gutter into the pelvis, with a component appearing loculated in the right lower quadrant just superior to the dome of the bladder. The pancreatic head was displaced anteriorly secondary to the complex fluid collection. No enlarged lymph nodes were identified. Abdominal ultrasound was ordered confirming free fluid with minimal debris and septations. The following day, interventional radiology conducted ultrasound-guided retroperitoneal collection drainage, which collected 750 mL of fluid, characteristic of aged blood. Fluid was sent for cytology, which revealed the presence of blood, negative for malignancy. Serum studies were negative for carcinoembryonic antigen (CEA), cancer antigen 19-9 (CA 19-9), and lactate dehydrogenase (LDH). The postoperative course was uncomplicated, and the patient was discharged on postoperative day one on fluconazole and combined amoxicillin-clavulanate potassium.

**Figure 2:**
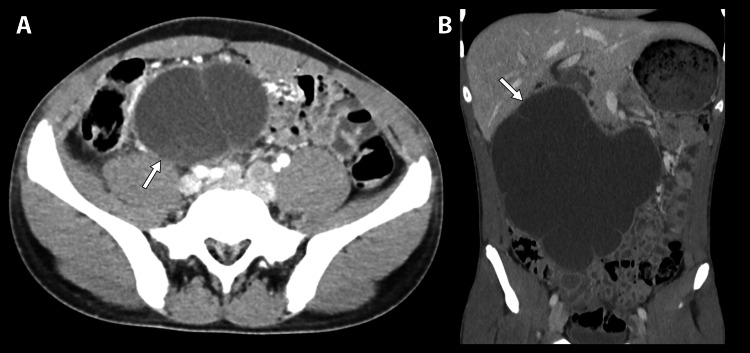
(A) Axial contrast-enhanced CT image showing a thin-walled cystic lesion in the right hemiabdomen. Note the lobulated contour with thin internal septal deviations. (B) Coronal contrast-enhanced CT imaging demonstrating the thin-walled cystic lesion measuring 8 x 10 x 19 cm along the transverse-anteroposterior-craniocaudal axis. The lobulated contour with thin internal septations is visualized.

Three months following discharge, the patient presented to the emergency department with a similar course to the previous presentation. Physical examination revealed a temperature of 36.9°C, a heart rate of 94 beats/minute, a respiration rate of 18 times/minute, a systolic and diastolic blood pressure of 119/67 mmHg, and a moderate right lower quadrant abdominal tenderness with no masses palpated (Table 2). A CT scan of the abdomen and pelvis with contrast was conducted revealing a large thin-walled cystic lesion in the right hemiabdomen containing minimal thin internal septations measuring 18 x 10 x 19 cm along the transverse-anteroposterior-craniocaudal axis (Figure 2). Trace fluid was identified in the pelvis. The mass resulted a moderate right hydronephrosis and hydroureter. No enlarged lymph nodes were identified. Laboratory results were notable for a white blood cell count of 11,300 with a left shift of 82.2%.

**Table 2:** Second admission vital signs and laboratory values.

Vital signs and laboratory values on the second admission		
Vital signs	Patient measurements	Normal range
Temperature	36.9°C	36.1°C-37.2°C
Heart rate	94 beats/minute	60-100 beats/minute
Respiratory rate	18 times/minute	12-20 times/minute
Systolic blood pressure	119 mmHg	90-120 mmHg
Diastolic blood pressure	67 mmHg	50-80 mmHg
Notable laboratory values		
White blood cell count	11,300/mm3	4,500-11,000/mm3
Neutrophil percentage	82.20%	32-58%
Absolute neutrophil count	9,300/mm3	2,500-6,000/mm3
Total bilirubin	0.26 mg/dL	0.1-1.0 mg/dL

**Figure 3:**
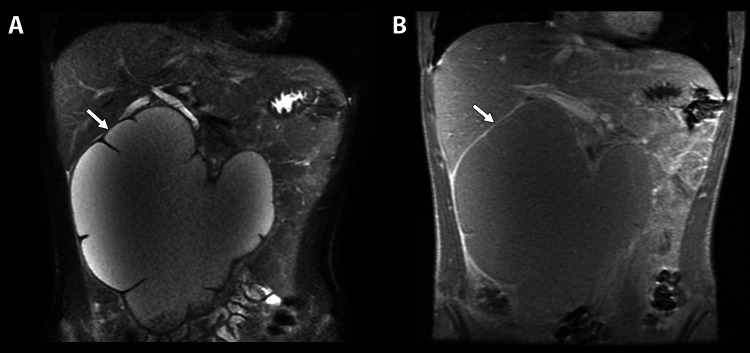
(A) Coronal T2-weighted single-shot fast spin echo (SSFSE) image demonstrating a large, lobulated, cystic mass containing internal septations along the periphery of the mass. (B) Coronal T1-weighted fast spoiled gradient-recalled echo (FSPGR) post-contrast image demonstrating the lobulated, cystic mass with visible septations and mild enhancement of the cyst wall.

Ultrasound of the genitals revealed moderate right hydrocele. Magnetic resonance imaging (MRI) with and without contrast was ordered to clarify the etiology and extent of the mass (Figure 3). Imaging results revealed a complex retroperitoneal mass within the right hemiabdomen that extends to the left of the midline. The mass measured 16.9 x 11.1 x 16.9 cm along the transverse-anteroposterior-craniocaudal axis, extending superiorly to the portal region and demonstrating a lobulated contour. The mass was homogeneous in its appearance, with increased T2 signal intensity and low to intermediate T1 signal intensity on the post-contrast images.

**Figure 4:**
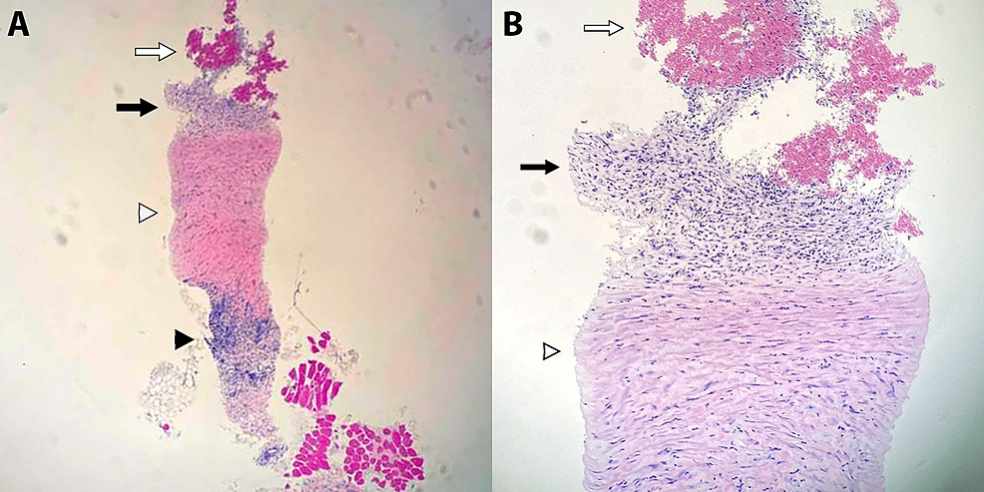
Microscopic section of CT core biopsy (hematoxylin & eosin). (A) Overview of the lymphatic malformation wall. The white arrow points to the intraluminal component containing aged blood. The black arrow points to a focus on organized fibrous connective tissue with reactive fibroblasts and aggregates of small lymphocytes. The white arrowhead points to benign connective tissue. The black arrowhead points to peripheral lymphoid aggregates (x10). (B) Higher resolution view of the probable cyst wall with the same identifiers as in image A. Note the focus of dense fibrosis forming the lymphatic malformation cyst wall as identified by the black arrow (x20).

The intraoperative course was eventful for one episode of diffuse abdominal pain necessitating 2 mg of morphine. A CT core biopsy of the retroperitoneal cyst wall and drainage was conducted with no operative complications. Drainage from the retroperitoneal collection resulted in 1,400 mL of aged, bloody fluid. CT biopsy results demonstrated portions of a probable cyst wall with organized fibers of connective tissue, containing reactive fibroblasts, nondescript aggregates of small lymphocytes, and hemosiderin macrophages (Figure 4). Due to the high likelihood of abdominal fluid collection recurrence, the patient was discharged on postoperative day one with a follow-up for outpatient sclerotherapy.

## Discussion

Lymphatic malformations are characterized by lymphatic vessel proliferation with resulting cystic structures due to interruption of lymphatic flow [6]. While the etiology of lymphatic malformations remains a discussed topic, two primary hypotheses have been identified. The current hypotheses consider congenital miscommunication of lymphatic cysts and blockage of lymphatic outflow, both highlighting the importance of balanced lymphatic flow for normal physiological presentation [7]. This plays an important role in considering lymphatic malformation regression, as continued interruption of lymph outflow may result in sustained lymphatic malformation expansion.

The clinical presentation of retroperitoneal lymphatic malformations is variable and often asymptomatic, which can make initial diagnoses challenging. Large cystic tumors in the retroperitoneum account for a wide list of differential diagnoses, including both benign and malignant lesions. Malignant lesions include germ cell tumors, undifferentiated sarcoma, necrotic neoplasms, cystic metastases, and malignant mesenchymoma, with benign lesions including pancreatic adenoma and pseudocysts, urothelial and foregut cysts, duplication cysts, and other masses such as retroperitoneal abscesses and hematomas [4,6,8]. In this patient's presentation, ascites may have been considered as part of the initial differential workup based on the initial CT abdominal free-fluid findings. Further evaluation reveals the retroperitoneal distribution of the fluid, especially as seen being posterior to the pancreas. This distribution does not follow the standard ascites fluid distribution, which lowers the likelihood of simple loculated ascites being present. The clinical symptoms of retroperitoneal lymphatic malformations often present with a palpable abdominal mass with associated mild abdominal pain but can be highly variable in presentation [1,4,5]. This was exemplified in this case, as the only notable clinical symptom observed was abdominal pain despite the large abdominal presence of the retroperitoneal lymphatic malformation. This highlights the difficulty in early diagnosis of retroperitoneal lymphatic malformations since the clinical presentation is variable and imaging cannot confidently rule out other etiologies without tissue pathology confirmation.

The appearance of lymphatic malformation on ultrasound presents as a well-defined, thin-walled, multilocular, anechoic cystic mass [6]. On CT imaging, lymphatic malformation typically presents as a homogeneous cystic mass that may have observable septal deviations, but can also present as a heterogeneous mass in the presence of blood, fat, or protein products [6]. Following intravenous contrast enhancement, lymphatic malformation may present a mild enhancement of the wall and septations on CT. MRI presentation of lymphatic malformation relies on the presence of internal products, such as blood, fat, and protein, but will typically have a homogeneous appearance with increased T2 signal intensity.

Treatment options for retroperitoneal lymphatic malformation include both surgical and conservative measures. Surgical excision is the first-line treatment for all retroperitoneal lymphatic malformations, due to low rates of tumor recurrence following complete resection [3]. However, complete excision may be challenging because of the infiltration properties of lymphatic malformations, often manifesting with poorly demarcated margins adjacent to vital structures [9]. For patients where surgical excision is not tolerable or advised, conservative methods may be considered, including aspiration and sclerotherapy. Aspiration promotes symptomatic relief, but the frequent reoccurrence of fluid collection may be expected, depending on the pathophysiology of the lymphatic malformation. Sclerotherapy involves aspiration of the lymphatic collection with concomitant usage of sclerosants, resulting in limited recurrence of the lymphatic malformation [9,10]. Both of the aforementioned conservative methods have been used in the patient presented here.

## Conclusions

In this case, we describe a unique presentation of retroperitoneal lymphatic malformation in a 15-year-old male. The patient’s clinical symptoms in both presentations were largely nonspecific, with abdominal pain being the primary finding. CT imaging on the initial presentation indicated a large fluid collection, absent of cystic components. Cystic components were visualized in the second presentation, having features reminiscent of a retroperitoneal lymphatic malformation. This portrays the challenge of early retroperitoneal lymphatic malformation diagnosis, as observed by their nonspecific clinical and imaging presentations. Confirmation of retroperitoneal lymphatic malformation is conducted through surgical exploration or tissue pathology, in which a tissue biopsy was protocoled in this presentation. Surgery is often necessary for retroperitoneal lymphatic malformation resolution; however, aspiration and sclerotherapy are viable non-surgical options.
